# Molecular and catalytic properties of fungal extracellular cellobiose dehydrogenase produced in prokaryotic and eukaryotic expression systems

**DOI:** 10.1186/s12934-017-0653-5

**Published:** 2017-02-28

**Authors:** Su Ma, Marita Preims, François Piumi, Lisa Kappel, Bernhard Seiboth, Eric Record, Daniel Kracher, Roland Ludwig

**Affiliations:** 1 Department of Food Sciences and Technology, Vienna Institute of Biotechnology, BOKU-University of Natural Resources and Life Sciences, Vienna, Austria; 2 UMR BDR, INRA, ENVA, Université Paris Saclay, 78350 Jouy en Josas, France; 3 Research Area Biochemical Technology, Institute of Chemical Engineering, TU Wien, Gumpendorferstrasse 1a, Vienna, Austria; 4 Aix Marseille Université, INRA, BBF (Biodiversité et Biotechnologie Fongiques), Marseille, France

**Keywords:** *Aspergillus niger*, Cellobiose dehydrogenase, Cofactor loading, *Escherichia coli*, Glycoforms, Heterologous expression, *Pichia pastoris*, *Trichoderma reesei*

## Abstract

**Background:**

Cellobiose dehydrogenase (CDH) is an extracellular enzyme produced by lignocellulolytic fungi. *cdh* gene expression is high in cellulose containing media, but relatively low CDH concentrations are found in the supernatant of fungal cultures due to strong binding to cellulose. Therefore, heterologous expression of CDH in *Pichia pastoris* was employed in the last 15 years, but the obtained enzymes were over glycosylated and had a reduced specific activity.

**Results:**

We compare the well-established CDH expression host *P. pastoris* with the less frequently used hosts *Escherichia coli*, *Aspergillus niger*, and *Trichoderma reesei*. The study evaluates the produced quantity and protein homogeneity of *Corynascus thermophilus* CDH in the culture supernatants, the purification, and finally compares the enzymes in regard to cofactor loading, glycosylation, catalytic constants and thermostability.

**Conclusions:**

Whereas *E. coli* could only express the catalytic dehydrogenase domain of CDH, all eukaryotic hosts could express full length CDH including the cytochrome domain. The CDH produced by *T. reesei* was most similar to the CDH originally isolated from the fungus *C. thermophilus* in regard to glycosylation, cofactor loading and catalytic constants. Under the tested experimental conditions the fungal expression hosts produce CDH of superior quality and uniformity compared to *P. pastoris*.

**Electronic supplementary material:**

The online version of this article (doi:10.1186/s12934-017-0653-5) contains supplementary material, which is available to authorized users.

## Background

Cellobiose dehydrogenase (CDH, EC 1.1.99.18, CAZy AA 3.1) is an extracellular flavocytochrome produced by a number of wood degrading fungi, when cellulosic materials are utilized as carbon source [1]. Recent studies showed that the physiological function of CDH is the donation of electrons to copper-dependent lytic polysaccharide monooxygenase (LPMO) [2, 3]. This oxidative CDH/LPMO system enhances the degradation rate of crystalline cellulose, and is widespread throughout the fungal kingdom together with the well-known hydrolytic cellulases [4, 5]. CDH is a monomeric enzyme that belongs to the glucose-methanol-choline (GMC) family of oxidoreductases [6]. It is composed of a large catalytic flavodehydrogenase domain (DH) containing one non-covalently bound flavin adenine dinucleotide (FAD). DH is connected to an electron transferring haem *b*-containing cytochrome domain (CYT) by a long, flexible linker enriched in hydroxy amino acids [7]. Recently reported crystal and solution structures of CDH demonstrated a dynamic interaction between DH and CYT. CYT acts as a mobile domain that reduces the active site copper of LPMO [7]. This intricate structure makes CDH a difficult enzyme to produce. A phylogenetic analysis of CDH sequences from various fungal sources showed a division of the enzymes into three distinct classes: class I represents only basidiomycetous CDHs, which are shorter in sequence and have a highly conserved linker sequence [8]; class II exclusively comprises the more complex ascomycetous CDHs, either with or without a type-1 carbohydrate-binding module, corresponding to classes IIA and IIB, respectively. Class III contains a different branch of so far uncharacterized CDHs [9]. Due to its electron transferring properties, CDH has been widely recognized as a versatile biorecognition element in electrochemical biosensors, which is capable of detecting a wide variety of carbohydrates (cellobiose, cellodextrins, lactose, maltose, glucose) as well as quinones and catecholamines [10, 11]. This ability is also exploited for the development of CDH-based anodes in enzymatic biofuel cells [12]. Because of the high interest in this oxidoreductase, high quality preparations of CDHs are requested in large quantities.

The production of CDHs by lignocellulose-degrading fungi results in reasonable amounts, especially in media containing pure cellulose [13]. However, the cellulose-binding ability of CDH results in a relatively low enzyme concentration in the supernatant, which makes the protein purification from fungal cultures difficult and time-consuming. Therefore, several *cdh* genes have been cloned and recombinantly expressed [14–16]. Recombinant protein production allows a fast, reliable and efficient enzyme production and the possibility to generate genetically engineered enzymes. *Escherichia coli* is one of the most commonly used industrial microorganisms, but the recombinant production of intact CDH has not been achieved for this host so far. Like other secreted eukaryotic proteins, CDH is subjected to posttranslational modifications that affect the properties of the mature protein significantly [17]. These posttranslational modifications, such as O- and N-linked glycosylation, are not introduced by *E. coli*. Only the DH domain of *Phanerochaete chrysosporium* was reported to be functionally expressed in *E. coli* [18]. The produced activity after cell lysis was 733 U L^−1^ and the enzyme was purified to a specific activity of 16.7 U mg^−1^ with a yield of 7.3%. *Pichia pastoris* has been used as a heterologous expression host for several basidiomycetous and ascomycetous CDHs [8, 14, 15, 19–21]. It typically achieves high expression levels, and well established strategies for large-scale production and easy genetic manipulation are available. The yeast performs basic eukaryotic post-translational modifications and typically secretes high amounts of functional enzymes. High expression levels were achieved for most CDHs produced by *P. pastoris*. In case of *Pycnoporus cinnabarinus* CDH, 7800 U L^−1^ (351 mg L^−1^) were measured in the culture supernatant [14]. It was, however, observed that CDHs produced by their natural hosts have a higher specific activity than CDHs recombinantly produced in *P. pastoris*. The specific activity, and consequently the turnover number, of recombinant CDH from *Corynascus thermophilus* (*Ct*CDH) was 5-times lower than observed for the enzyme isolated from the fungus [15]. This discrepancy was caused by a sub-stoichiometric occupation of catalytic sites with the FAD cofactor. Furthermore, recombinant CDHs produced by *P. pastoris* are typically over- or hyper-glycosylated, which might affect the essential intramolecular electron transfer reaction. To avoid these shortcomings, an expression host more closely related to fungal CDH producers would be of advantage. Nowadays, filamentous fungi are also well developed as expression hosts that are able to secrete large amounts of target proteins [22]. The development of new molecular genetic tools facilitates the usage of fungi in protein production [23], although only a limited number of fungal species have been explored so far, such as *Aspergillus niger*, *A. oryzae* or *Trichoderma reesei*. To date, two CDHs have been recombinantly produced in *A. oryzae* [24] and two in *A. niger* [16]. Maximal CDH activity reached 7620 U L^−1^ for the basidiomycetous *Coprinopsis cinerea* CDH, whereas only 126 U L^−1^ were measured for the ascomycetous *Podospora anserina* CDH [16]. However, the quality of the recombinant enzyme in comparison to *P. pastoris* derived CDH cannot be judged due to the limited information. Wang and Lu heterologously expressed the *cdh* gene from *P. chrysosporium* in *T. reesei* to study the synergism between CDH and cellulases [25]. But there is no further data of enzyme production or enzyme characterization. In order to identify the best expression host for high quality recombinant CDH, a detailed comparison of enzymes produced by the different expression hosts is required.

It was the objective of this study to compare four expression systems in regard to their recombinantly produced DH/CDH from *Corynascus thermophilus* (*Ct*CDH). The prokaryotic expression system *E. coli* was explored to recombinantly produce the catalytic dehydrogenase domain of *Ct*CDH (*Ct*DH). The intact, full-length *Ct*CDH was expressed in *P. pastoris* and in the two filamentous fungi *A. niger* and *T. reesei*. In order to achieve high productivity, all cultivations were carried out in a scalable bioreactor system. Finally, the recombinant enzymes were purified and their spectral properties, uniformity of glycosylation, kinetic constants and thermostability were compared.

## Results

### Production of the *Ct*DH domain in *E. coli* and chromatographic purification

**Fig. 1:**
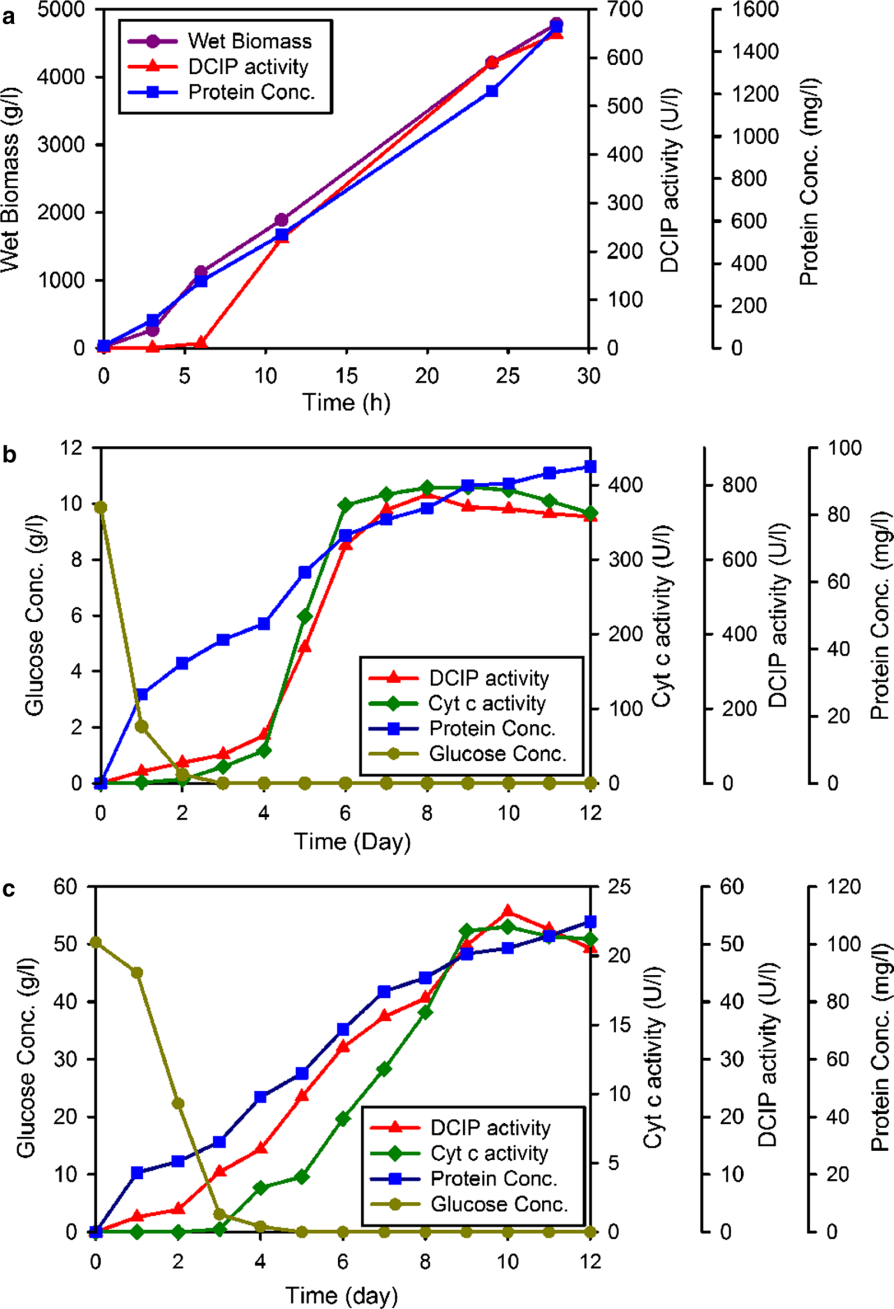
Production of *Ct*DH/*Ct*CDHs expressed in *E. coli* (**a**), *A. niger* (**b**) and *T. reesei* (**c**). *Purple circles* wet biomass; *red triangles* volumetric activity (DCIP assay at acetate buffer pH 5.5), *green diamonds* volumetric activity (cyt *c* assay in Tris/HCl buffer, pH 7.5), *blue squares* protein concentration (per broth volume), *dark yellow circles* glucose concentration of fermentation broth. The measurements were done in triplicates; the difference between the values was less than 5%

Production of *Ct*DH was carried out in a 7-L bioreactor. In order to increase protein solubility, the operating temperature was reduced from 30 to 25 °C at an OD_600_ of 0.65. The fermentation was stopped after 28 h when the specific activity started to decline. The highest volumetric activity obtained was 648 U L^−1^ (Fig. 1a) with a corresponding specific activity of 0.42 U mg^−1^. Approx. 1.5% (w/w) of the total proteins in the cell lysate accounted for *Ct*DH.

The fermentation broth was harvested at a wet biomass concentration of approx. 4800 g L^−1^ and was disrupted using a homogenizer. After a one-step chromatographic purification, electrophoretic analysis showed that three major bands at approximately 53, 25 and 12 kDa were present (Additional file 1: Figure S1). Separation of these proteins was achieved by an ultrafiltration step. *Ct*DH with a molecular mass of 53 kDa was purified to homogeneity at a yield of 60% and had a specific activity of 27.5 U mg^−1^ (Table 1). A summary of the purification procedures is presented in Additional file 1: Table S1. After purification, a bright-yellow protein solution typical for flavoproteins was obtained.

**Table 1:** Comparison of enzyme production and purification for the four expression hosts

DH/CDH expressed by	*E. coli*	*P. pastoris* [15]	*A. niger*	*T. reesei*
Enzyme production				
Cultivation time (h)	28	111	288	288
Wet cell mass (g L^−1^)	4785 ± 160	275	n.d.	n.d.
Soluble protein conc. (mg L^−1^)	1517 ± 44^a^	633	108 ± 4	94 ± 3
DCIP activity (U L^−1^)	648 ± 19	376	49 ± 2	715 ± 14
Cyt *c* activity (U L^−1^)	–	320	21 ± 1	362 ± 11

DH/CDH expressed by	*E. coli*	*P. pastoris*	*A. niger*	*T. reesei*
Enzyme purification				
Purification steps	IMAC	Phenyl Sepharose and QSource	Phenyl Sepharose and QSource	Phenyl Sepharose and QSource
Purification yield (%)	60	71	54	58
Purification (fold)	65.5	7.7	51.8	3.4
Specific activity for DCIP (U mg^−1^)	27.5 ± 0.3	9.4 ± 0.2	14.1 ± 0.1	14.3 ± 0.1
Specific activity for cyt *c* (U mg^−1^)	–	3.5 ± 0.1	11.1 ± 0.2	12.5 ± 0.2
FAD loading (%)	52 ± 2	44 ± 1	56 ± 1	68 ± 3
Glycosylation (%)	0	18.9 ± 12.1	9.4 ± 2.7	1.4 ± 1.3

The measurements were done in triplicates

*n.d.* not determined, – the DH domain has no cyt *c* activity

^a^Intracellular protein

### Production of *Ct*CDH in *P. pastoris* and purification

Production of *Ct*CDH in *P. pastoris* was carried out in a 7-L bioreactor according to a previously published protocol [15]. The volumetric CDH activity in the culture supernatant reached 376 and 320 U L^−1^ when measured with the DCIP assay and the cyt *c* assay, respectively. The recombinant CDH constituted up to 14% of the total proteins in the fermentation broth. *Ct*CDH with an average molecular mass of 94 kDa was purified to homogeneity resulting in a specific activity of 9.4 U mg^−1^ (DCIP assay) and 3.5 U mg^−1^ (cyt *c* assay) at a final yield of 71%.

### Production of *Ct*CDH in *A. niger* and purification

Transformants were selected for their ability to grow on minimal medium plates without uridine. Approximately 100 uridine prototrophic transformants were obtained per microgram of expression vector and 24% of the obtained transformants showed CDH activity in the extracellular media after 10 days of cultivation.

The transformant with the highest *Ct*CDH production was selected for further experiments. Production was carried out in a 7-L bioreactor with 4 L of *A. niger* culture medium inoculated with 8 × 10^8^ asexual spores. The glucose concentration dropped below the measurable concentration after the first 3 days and fast growth of *A. niger* mycelium was observed before day 3. CDH activity increased gradually from day 3 to day 9, and then reached its peak activity. The culture supernatant showed a volumetric activity of 49 U L ^−1^ with the DCIP assay and 21 U L^−1^ with the cyt *c* assay at day 10 (Fig. 1b). The specific activity of the fermentation broth prior to purification was 0.44 U mg^−1^ with the DCIP assay and 0.19 U mg^−1^ with the cyt *c* assay. The recombinant CDH made up 3.5% of the total protein mass in the fermentation broth.

The recombinant enzyme was purified to homogeneity using a two-step purification protocol employing hydrophobic interaction chromatography and anion exchange chromatography. The purity of *Ct*CDH increased 52-fold and the final purification yield was 54%. The specific activity of the homogeneous enzyme was 14.1 U mg^−1^ with the DCIP assay and 11.1 U mg^−1^ with the cyt *c* assay.

### Production of *Ct*CDH in *T. reesei* and purification

Transformants were selected based on hygromycin resistance and purified to uninuclear clones through a single-spore culture. Twenty transformants that grew well on hygromycin plates were selected for CDH expression in shaking flask cultures. To scale-up enzyme production, the highest producing clone of *T. reesei* was cultivated in a 2-L bioreactor. The glucose concentration decreased by 80% after the first day of incubation. The CDH activity increased rapidly from day 4 to 6, when full depletion of glucose was observed. The peak activity reached 715 U L^−1^ with the DCIP assay and 362 U L^−1^ with the cyt *c* assay (Fig. 1c). No further increase of CDH activity was observed from day 6 to 12, which suggests that the expression stopped at day 6 due to lack of glucose. The *Ct*CDH concentration in the crude supernatant reached 29 mg L^−1^ based on the specific activity of the purified enzyme.

The recombinant *Ct*CDH expressed in *T. reesei* was purified to apparent homogeneity as described for *A. niger*, and resulted in a 3.4-fold purification with a yield of 58% (Table 1). The already high specific activity of the culture supernatant (3.7 U mg^−1^) indicates that the recombinant *Ct*CDH is the main secreted protein (30%) in the *T. reesei* fermentation broth. The specific activity of the homogeneous enzyme was 14.3 U mg^−1^ with the DCIP assay and 12.5 U mg^−1^ with the cyt *c* assay.

### Characterization of recombinant *Ct*DH and *Ct*CDHs

#### Molecular properties

**Fig. 2:**
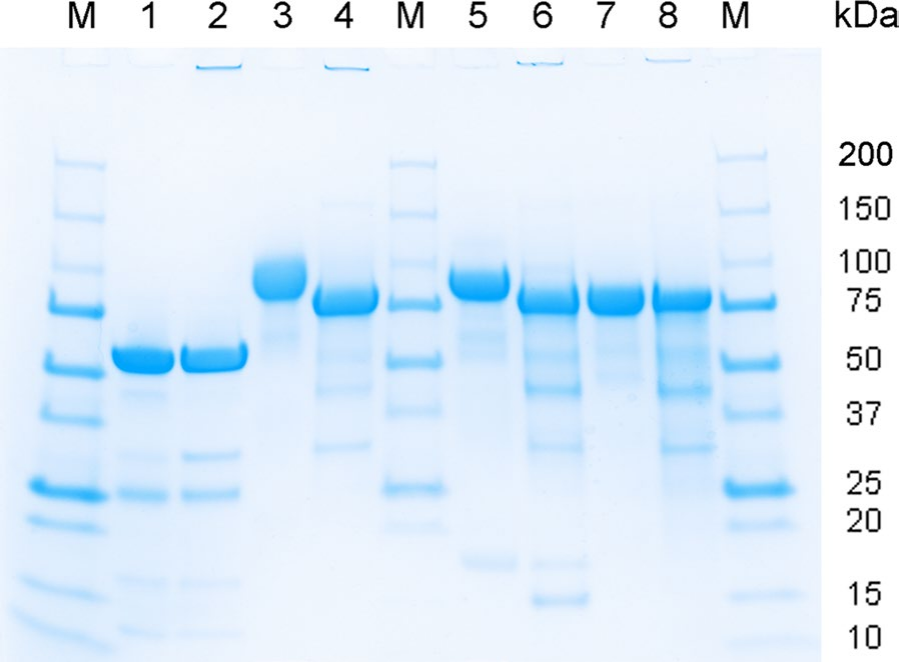
SDS-PAGE of purified and deglycosylated *Ct*DH or *Ct*CDHs. *Lane M* Precision Plus Protein Dual Color Standards (Bio-rad), *lane 1* purified *Ct*DH expressed in *E. coli*, *lane 2* deglycosylated *Ct*DH expressed in *E. coli*, *lane 3* purified *Ct*CDH expressed in *P. pastoris*, *lane 4* deglycosylated *Ct*CDH expressed in *P. pastoris*, *lane 5* purified *Ct*CDH expressed in *A. niger*, *lane 6* deglycosylated *Ct*CDH expressed in *A. niger*, *lane 7* purified *Ct*CDH expressed in *T. reesei*, *lane 8* deglycosylated *Ct*CDH expressed in *T. reesei*

Molecular masses of all recombinant enzymes were determined by SDS-PAGE (Fig. 2). The *Ct*CDH expressed in *P. pastoris* showed a broad and diffuse band between 80 and 98 kDa, which was likely caused by heterogeneous glycosylation and the smearing of glycoproteins on SDS-PAGE gels, whereas the *Ct*CDH expressed in *A. niger* showed a smaller and sharper band (82 ± 2 kDa). The *Ct*CDH expressed in *T. reesei* showed the sharpest band with a molecular mass of 76 ± 1 kDa, indicating little glycosylation and a low heterogeneity of the protein. After deglycosylation under denaturing conditions with PNGase F, single sharp bands with an identical molecular mass of 75 kDa were found for all CDHs. The *Ct*DH expressed in *E. coli*, before and after deglycosylation, showed identical bands with a molecular weight of 53 kDa. The additional bands at 35 kDa in the deglycosylated samples originate from PNGase F.

**Fig. 3:**
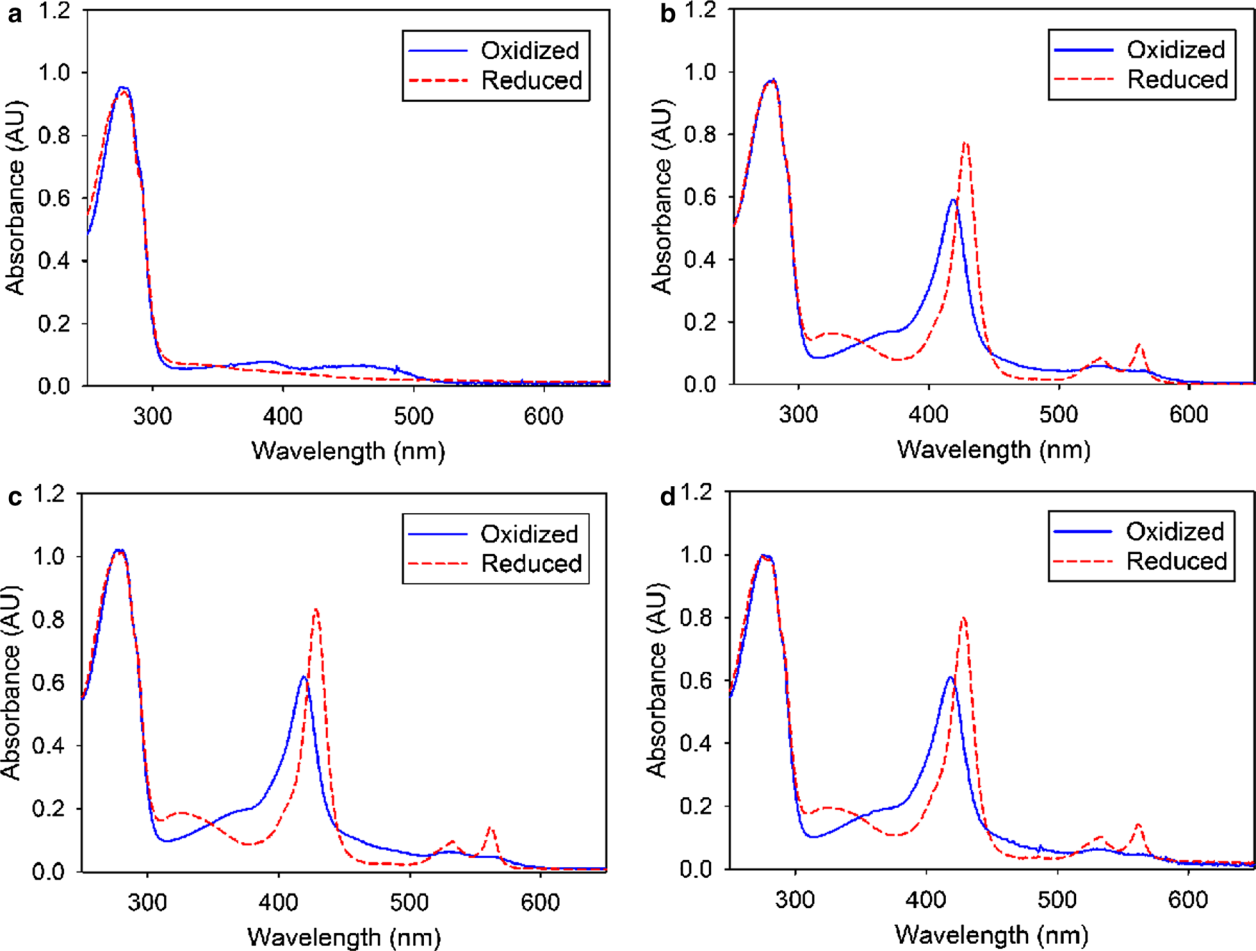
Spectral characterization of *Ct*DH/*Ct*CDHs showing the oxidized (*blue line*) and reduced (*red dashes*) spectra. Some grains of solid cellobiose were added to the cuvette and used for reduction. Spectra of *Ct*DH or *Ct*CDHs expressed in *E. coli* (**a**), *P. pastoris* (**b**), *A. niger* (**c**) and *T. reesei* (**d**) are shown

The UV/Vis spectrum of the purified *Ct*DH (Fig. 3a) is characteristic for a flavoprotein. The FAD cofactor has an absorption spectrum with a maximum at 450 nm and a wide shoulder in the region of 360 nm. The FAD spectra of full-length CDHs are partially overlaid by the haem *b* absorbance. The spectra of all purified *Ct*CDHs (Fig. 3b–d) show the typical characteristics of a flavocytochrome. The major peak of the oxidised spectrum at 420 nm is the Soret peak of the haem cofactor, whereas the broad shoulder between 450 and 500 nm is mainly attributed to the FAD cofactor. Upon reduction of *Ct*CDH by its native substrate cellobiose, peaks appeared at 429, 533, and 564 nm, representing the Soret-, β- and α- peaks of the reduced haem. In this state, the absorption in the region between 450 and 500 nm decreased due to reduction of the FAD. The R_Z_ (A_420_/A_280_) values for *Ct*CDHs expressed in *P. pastoris*, *A. niger* and *T. reesei* were 0.60, 0.61 and 0.61, respectively, indicating the same, high purity. After protein precipitation with trichloroacetic acid [26], the amount of the released FAD was determined. We found that 52% of the active sites of the DH domain expressed in *E. coli* contained FAD and that the FAD loading of *Ct*CDH expressed in *P. pastoris*, *A. niger* and *T. reesei* was 44, 56 and 68%, respectively.

#### Catalytic properties

The catalytic constants for the reduction of the two-electron acceptor DCIP were determined at pH 5.5, whereas reduction of the one-electron acceptor cyt *c* was determined at pH 7.5 (Table 2). The K_M_ values for cellobiose were similar for all enzymes, and were found to be around 362 µM measured with DCIP as electron acceptor and 100 µM using cyt *c* as electron acceptor. In contrast to the almost identical K_M_ values, a systematic difference of the *k*_*cat*_ values among the *Ct*DH and the three *Ct*CDHs was observed. The *Ct*DH expressed in *E. coli* had the highest *k*_*cat*_ value (15.5 s^−1^) for DCIP. *Ct*CDH expressed by the eukaryotic hosts *T. reesei* and *A. niger* showed also high *k*_*cat*_ values of 15.2 and 13.3 s^−1^, respectively, but *Ct*CDH expressed in *P. pastoris* had a twofold lower *k*_*cat*_. The cyt *c* activity for both filamentous fungal expression systems was equally good, resulting in *k*_*cat*_ values of 7.6 and 7.9 s^−1^. For *P. pastoris* the turnover number was 3 times lower.

**Table 2:** Comparison of steady-state kinetic constants of *Ct*DH and *Ct*CDHs for cellobiose

Enzyme	K_M_ (μM)	*k*_*cat*_ (s^−1^)	*k*_*cat*_/K_M_ (mM^−1^ s^−1^)
DCIP assay			
*E. coli* (DH)	367 ± 11	15.5 ± 0.2	42
*P. pastoris* (CDH)	373 ± 14	8.1 ± 0.3	22
*A. niger* (CDH)	357 ± 18	13.3 ± 0.3	37
*T. reesei* (CDH)	349 ± 21	15.2 ± 0.4	44
Cyt *c* assay			
*P. pastoris* (CDH)	81 ± 7	2.6 ± 0.1	32
*A. niger* (CDH)	102 ± 5	7.6 ± 0.2	74
*T. reesei* (CDH)	118 ± 11	7.9 ± 0.2	67

The measurements were done in triplicates

#### Thermostability

The thermostability of *Ct*DH and *Ct*CDHs was determined using the *Thermo*FAD method [27] and the intrinsic tryptophan fluorescence upon protein unfolding. All enzymes showed transition midpoint temperatures (T_m_) in a narrow temperature range of 58.1 ± 1.5 °C. T_m_ values obtained with the *Thermo*FAD method were generally higher by 1–1.5 °C than T_m_ values obtained from tryptophan fluorescence (Table 3; Additional file 1: Figure S2). The T_m_ values for *Ct*CDH expressed in *P. pastoris* and *A. niger* were slightly higher than those for *Ct*CDH expressed in *E. coli* and *T. reesei*. This was observed with both methods. Protein precipitation occurred at the end of the unfolding experiments in case of the non-glycosylated *Ct*DH and the less glycosylated *Ct*CDHs expressed by *A. niger* and *T. reesei*.

**Table 3:** Transition midpoint temperatures (*T*_m_) measured with the *Thermo*FAD method and the intrinsic tryptophan fluorescence

Enzyme	*Thermo*FAD	Trp unfolding
*E. coli* (DH)	57.7 ± 0.3	56.9 ± 0.4
*P. pastoris* (CDH)	59.2 ± 0.1	57.4 ± 0.6
*A. niger* (CDH)	59.7 ± 0.1	57.5 ± 0.5
*T. reesei* (CDH)	57.8 ± 0.2	56.6 ± 0.3

The measurements were done in triplicates

## Discussion

This study compared the suitability of four different expression systems for the production of CDH. We evaluated the titer of secreted CDH, the purification procedure and yield together with the molecular and kinetic properties of the purified, recombinant enzymes. The prokaryotic expression host *E. coli* can express only the DH domain of *Ct*CDH, but produced a high amount of it in a much shorter time (28 h) than the tested eukaryotic expression systems. Per liter of fermentation medium 652 U of CDH activity were obtained, which is 1.7-fold higher than the previously reported amount of the DH domain from *Phanerochaete chrysosporium* (*Pc*DH) expressed in *E. coli* [18].

The full-length CDH, comprising both CYT and DH, could so far only be functionally expressed in eukaryotic expression hosts. Several CDHs have been successfully expressed in the methylotrophic yeast *P. pastoris*, including *Ct*CDH [15]. The reported volumetric expression levels vary from 376 to 7800 U L^−1^, which can be recalculated to CDH concentrations between 79  and 351 mg L^−1^, respectively (Additional file 1: Table S2). Four CDHs have been expressed in the fungal expression hosts *A. oryzae* and *A. niger* [16, 24], with different expression levels. Additional file 1: Table S2 shows that basidiomycetous CDHs are generally expressed with higher volumetric activities than ascomycetous CDHs, which typically have lower specific activities. In terms of produced CDH concentration, the expression levels between basidiomycetous and ascomycetous CDHs are comparable, but differences arising from diverse fermentation protocols are obvious. In this work, the production level of *Ct*CDH in *A. niger* was very low (55 U L^−1^), which is in accordance with the reported data for other ascomycetous CDHs. This study reports also the first successful expression of *Ct*CDH in *T. reesei*. The expression level of 800 U L^−1^ was the highest among the tested eukaryotic expression hosts in this study and compares well with other expression hosts in terms of secreted CDH concentration (32 mg L^−1^). Since *T. reesei* is known to produce high concentrations of recombinant proteins, an optimization of the fermentation protocol should further increase the CDH yield [29].

The specific CDH activity in the cell lysate or the culture supernatant differed among the expression hosts and influenced the purification strategy. The weight percentage of *Ct*DH in the *E. coli* cell lysate was only 1.5% and a high amount of intracellular proteins was present. For easy purification of the DH domain it was necessary to fuse a C-terminal His-tag to the enzyme, which allowed a one-step purification via immobilized metal affinity chromatography and resulted in a much higher yield of 60% compared to only 2% for recombinant *Pc*DH without His-tag [18]. Although *E. coli* is not able to introduce eukaryotic posttranslational modifications, the homogeneous *Ct*DH exhibited a high specific activity of 27.5 U mg^−1^ using the DCIP assay.

*Pichia pastoris* is a well-established expression system which allows for a relatively high *Ct*CDH production (15% of total protein). However, the low cofactor occupancy and overglycosylation resulted in a *Ct*CDH with a limited quality. The specific activity was 9.4 U mg^−1^ for DCIP and 5.3 U mg^−1^ for cyt *c*. Fungal hosts are thus preferable for the expression of high quality *Ct*CDH. Homogenous *Ct*CDH has a specific activity around 14 U mg^−1^ using the DCIP assay and 12 U mg^−1^ using the cyt *c* assay, which is comparable to the enzyme expressed by the native host [15]. The newly employed expression host *T. reesei* showed the capability of secreting the mature *Ct*CDH into the fermentation broth, which made up 30% of all proteins in the supernatant. *A. niger* gave a low expression level (3.5%) for this ascomycetous CDH. The purification strategy worked similarly efficient for all three eukaryotic expression systems, giving yields between 54 and 60%.

Glycosylation is a common post-translational modification in extracellular fungal proteins. Presumably, all native CDHs contain glycan structures, although the degree of glycosylation varies significantly, from 2% [28] up to 15% [29] of the total CDH protein mass. However, the most commonly used expression host *P. pastoris* usually attaches high mannose structures as N-glycosides, which can make up for 10–48% of the total mass (Additional file 1: Table S2). Six predicted glycosylation sites are found for the intact *Ct*CDH using NetNGlyc 1.0, of which five are surface exposed and located on the DH domain. Interestingly, three glycosylation sites are close to the linker connecting DH and CYT. In addition, there are eleven putative O-glycosylation sites located on both domains (NetOGlyc 4.0). However the extent of O-glycosylation in native CDHs is uncertain. We conclude that the demand for highly active and uniform recombinant CDH preparations used for electrochemical and biocatalytic applications might be best served by CDH produced by *T. reesei*. SDS-PAGE indicated a very uniform glycosylation that makes up approx. 1.3% of the total protein mass. A higher percentage of glycosyl residues was observed for *Ct*CDH expressed in *A. niger* (8%) and the overglycosylation and heterogeneity of *Ct*CDH produced in *P. pastoris* is least favorable in this respect (7–30%).

Several sequence and structural factors have been proposed to contribute toward a greater stability of thermophilic proteins, such as the presence of prolines in loop regions or the stability of alpha-helix and surface salt bridges [30–32]. Glycosylation is another feature of eukaryotic proteins that frequently contributes to stability [33]. We compared the melting temperature of *Ct*DH and all *Ct*CDHs expressed in the study. Interestingly, neither the different FAD occupancy of the enzyme preparations nor their different degree of glycosylation influenced the overall thermostability. Using two methods, we showed that the T_m_ values of all enzyme preparations were within 1.5 °C, including the least flavinated *Ct*CDH expressed in *P. pastoris*.

The presence of the FAD cofactor in the DH domain of CDH is crucial for the enzymatic activity and was reported as a limiting factor in *P. pastoris* expressed *Ct*CDH [15]. In this work, the FAD loading of all four enzymes was experimentally measured and analyzed together with the catalytic turnover. For eukaryotic expression hosts, *Ct*CDH expressed in *T. reesei* had the highest FAD loading (65%) and gave the highest turnover number. In contrast, *Ct*CDH expressed in *P. pastoris* showed the lowest specific activity and the lowest FAD content. The re-calculated *k*_*cat*_ values (normalized by the FAD loading) are very similar (21.5 ± 2.8 s^−1^), demonstrating that the FAD loading is the key factor for the catalytic capability of the DH domain. However, the re-calculated *k*_*cat*_ value of *Ct*DH is higher (29.8 s^−1^), which could indicate that the substrate channel is more accessible in absence of the CYT domain.

The DCIP reduction rate depends only on the catalytic reaction in the DH domain, whereas the reduction of cyt *c* is limited by the intramolecular electron transfer (IET) between DH and CYT. The cyt *c* assay has been reported to give a good estimation of the IET rate, because the electron transfer between the cofactors is, compared to the catalytic turnover at the FAD, rate limiting. It is interesting to observe that overglycosylation of the CDH molecule reduces the IET rate. A decrease of the IET is visible from the 3–5 times lower *k*_*cat*_ values of cyt *c* reduction for the highly glycosylated *Ct*CDH expressed by *P. pastoris* and *A. niger* (Table 2). The *k*_*cat*_ value of *Ct*CDH expressed in *A. niger* for cellobiose measured with the DCIP assay is about two times higher than for *Ct*CDH expressed in *P. pastoris* whereas the *k*_*cat*_ value for cellobiose measured with the cyt *c* assay is two times lower. This indicates that the *Ct*CDH expressed in *A. niger* has a reduced IET rate. A comparison of the specific cyt *c* activity of the recombinant *Ct*CDH expressed by *T. reesei* with *Ct*CDH produced by *C. thermophilus* show a similar value, but the specific activity for DCIP is only 40% compared to the published data [15]. These results suggest that for recombinant CDH expressed by fungal hosts, the FAD cofactor loading is a more important issue than glycosylation. An optimization of CDH expression in *T. reesei* seems to be the best starting point for future studies.

## Conclusions

*Escherichia coli* is a good expression system to express the flavodehydrogenase domain of *Ct*CDH. It is easy to manipulate and fast producing. The *P. pastoris* expression system allows high protein yields, however, the low FAD loading and overglycosylation of CDH cause low specific activities. The fungal expression systems produced *Ct*CDH of superior quality and uniformity. Under the tested experimental conditions, the production of *Ct*CDH by *A. niger* results in lower amounts than *Ct*CDH production by *T. reesei*, but both fungal expression systems could be further optimized towards increased productivity by optimizing promoters and expression conditions. In conclusion, *T. reesei* is the best expression system for recombinant *Ct*CDH production. The produced *Ct*CDH has a high cofactor loading and the glycosylation and specific activity are closest to the CDH isolated from *C. thermophilus*.

## Methods

### Strains and media

The chemically competent *E. coli* strains NEB 5α and T7 Express were purchased from New England Biolabs (New England BioLabs, Frankfurt, Germany). *E. coli* NEB 5α was used for vector construction and propagation. *E. coli* cells were grown at 37 °C in lysogeny broth (LB) or on LB agar supplemented with ampicillin (100 mg mL^−1^). MagicMedia *E. coli* expression medium (Thermo Fisher Scientific) was used for expression studies.

*Pichia pastoris* strain X-33 containing the published plasmid pPICctcdh was used for *Ct*CDH expression [15]. *P. pastoris* cells were grown in yeast peptone dextrose (YPD) broth or on YPD plates with zeocin (100 mg L^−1^) at 30 °C and the Basal Salts Medium was used for fermentation.

**Fig. 4:**
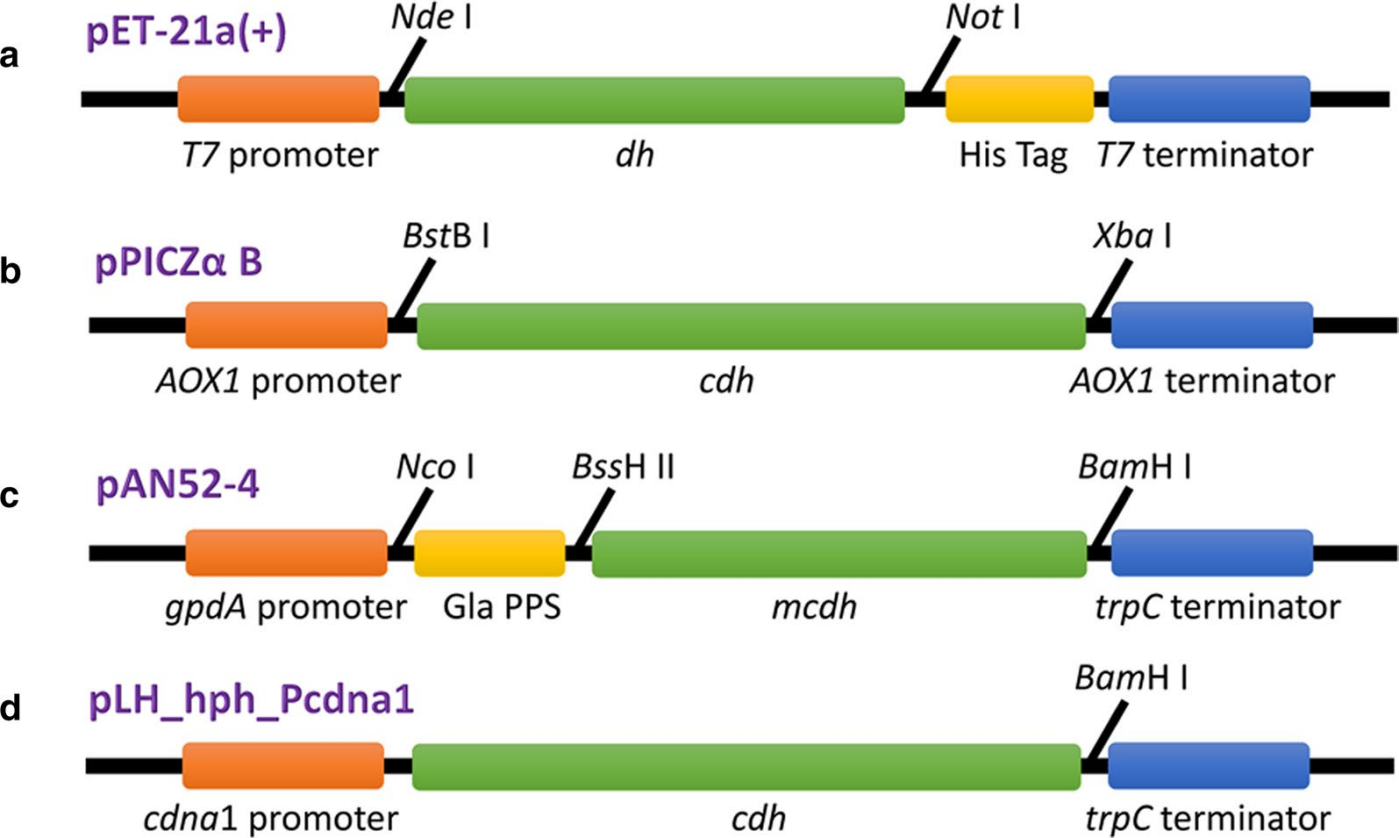
Expression plasmids for *Ct*DH/*Ct*CDHs production. Schematic presentation of the *Ct*DH/*Ct*CDHs expression cassettes used in this study. *dh* the encoding gene of the flavin domain of *Ct*CDH, *cdh* the encoding gene of the full-length of *Ct*CDH with its own signal peptide, *mcdh* the encoding gene of the mature *Ct*CDH without its own signal peptide, *Gla PPS* the gene of the glucoamylase (GLA) prepro sequence. See experimental procedures for more information

*Aspergillus niger* strain D15#26 (*pyrG*-) [34] was used for heterologous expression of the recombinant *Ct*CDH. After cotransformation with vectors containing the *pyrG* gene and the expression cassette (Fig. 4c), respectively, transformants of *A. niger* were selected at 30 °C on solid minimal medium (without uridine) containing 70 mM NaNO_3_, 7 mM KCl, 11 mM KH_2_HPO_4_, 2 mM MgSO4, and 1% (w/v) glucose and trace elements (1000× stock; 76 mM ZnSO_4_, 178 mM H_3_BO_3_, 25 mM MnCl_2_, 18 mM FeSO_4_, 7.1 mM CoCl_2_, 6.4 mM CuSO_4_, 6.2 mM Na_2_MoO_4_, and 174 mM EDTA). The best producing transformant for CDH expression was screened on culture medium containing 70 mM NaNO_3_, 7 mM KCl, 200 mM Na_2_HPO_4_, 2 mM MgSO_4_, 5% (w/v) glucose and trace elements at pH 5.5 inoculating it with 2 × 10^6^ spores mL^−1^. The same medium was used for fermentation.

*Trichoderma reesei Δxyr1* strain [35] was used for *Ct*CDH expression. This strain derived from *T. reesei* QM9414 (ATCC 26921) is deleted in the major cellulase and xylanase regulator which provides a (hemi)cellulase free background. It was maintained on potato dextrose agar (PDA) plates at 28 °C. Transformants of *T. reesei* were grown for selection on PDA with hygromycin B (50 mg L^−1^). For *Ct*CDH expression, strains were grown at 28 °C in a modified MA-Medium (Mandels–Andreotti) [36] containing 10 g L^−1^ glucose, 1.4 g L^−1^ (NH_4_)_2_SO_4_, 2.0 g L^−1^ KH_2_PO_4_, 0.3 g L^−1^ MgSO_4_·7H_2_O, 0.4 g L^−1^ CaCl_2_·2H_2_O, 1 g L^−1^ peptone, and 1/50 (v/v) of the trace element solution (0.25 g L^−1^ FeSO_4_·7H_2_O, 0.08 g L^−1^ MnSO_4_·H_2_O, 0.07 g L^−1^ ZnSO_4_·7H_2_O, 0.1 g L^−1^ CoCl_2_·2H_2_O) at pH 5.0 inoculated with 10^6^ spores mL^−1^. The same medium was used for fermentation.

### Chemicals and vectors

All chemicals were purchased from Sigma, Fluka, Roth or VWR and were of the highest purity available. Primers were synthesized by Microsynth and nucleotide sequences are shown in Table 4. Restriction enzymes, dNTP mix and T4 DNA ligase were from Fermentas and the Phusion polymerase from New England Biolabs. The plasmid pET-21a (+) from Novagen was used for expression in *E. coli* under control of the T7 promoter. For *Ct*CDH expression in *A. niger,* pAN52-4 [37] and pAB4-1 [38] containing the *pyrG* selection marker were used. In expression vector pAN52-4, the *A. niger* constitutive *gpdA* promoter and the *A. nidulans trpC* terminator were used to drive the expression of recombinant *Ct*CDH. Vector pLH_hph_Pcdna1 [39] containing an hygromycin B expression cassette as fungal selection marker followed by the constitutive *cdna*1 promoter region was used for *Ct*CDH expression in *T. reesei*.

**Table 4:** Nucleotide sequences of primers used in this study

Primer name	Sequence (from 5′ to 3′)
Pecoli-F1	GGAATTCCATATGGACACGTATGATTACATCGT
Pecoli-R1	ATAAGAATGCGGCCGCATAACGCAGGGACAGGATGC
Paniger-F1	TTGGCGCGCTCAGATGACCGAAGGGACGTA
Paniger-R1	CGGGATCCCTAATACCGCAGGGACAGGA
Ptreesei-F1	TCATCGATGTCGACCATGAAGCTTCTCAGCCGCGTTG
Ptreesei-R1	CGGGATCCCTAATACCGCAGGGACAGGATG
Ptrpc-F1	CGGGATCCGAAGCTTGAGATCCAC
Ptrpc-R1	CCAAGCTTGCATGCCAAGAAGGATTACCTCTAAACAAG

The underlined characters indicate the restriction site

### Construction of the expression vectors

The gene of the CDH flavin domain was PCR-amplified from pPIC*ctcdh* (Fig. 4b), a vector previously created to express the entire CDH in *P. pastoris* under the control of the AOX1 promoter [15]. Primers Pecoli-F1 and Pecoli-R1 (Table 4) were used to introduce the *Nde*I and *Not*I restriction sites, respectively. The PCR product was purified, digested and ligated into the 5′-*Nde*I and 3′-*Not*I sites of pET-21a(+) in-frame with the six-histidine tag to generate plasmid pET21-CtDH (Fig. 4a).

The DNA fragment encoding the mature *Ct*CDH was amplified using primers Paniger-F1 and Paniger-R1 (Table 4). The amplicon was integrated into the pAN52-4 expression vector using a restriction cloning approach with *Bss*HII and *Bam*HI enzymes. In addition, the synthesized oligonucleotides of the glucoamylase (GLA) prepro sequence [40] was digested with *Nco*I and *Bss*HII and ligated into the respective sites of pAN52-4 using the Rapid DNA Ligation Kit from Fermentas (Fig. 4c). The final construct pAN52-CtCDH was used to express *Ct*CDH in *A. niger*.

The gene encoding the full-length CDH with its own signal peptide was PCR-amplified from pPIC*ctcdh* using primers Ptreesei-F1 and Ptreesei-R1 (Table 4). A PCR-amplicon of the *A. nidulans trpC* terminator was digested by *Bam*HI and ligated with the same predigested PCR product of *Ct*CDH. The vector pLH_hph_Pcdna1 was linearized by *Sbf*I restriction enzyme and ligated with DNA fragment of *Ct*CDH and TrpC terminator using In-Fusion HD Cloning Kits following the manufacturer’s protocol (Takara Bio Europe, Saint-Germain-en-Laye, France) (Fig. 4d). Correct insertion of the gene was checked by DNA sequencing.

### Selection of the production clones

Correct insertion of the *Ct*DH gene was checked by DNA sequencing and verified plasmid was transformed into *E. coli* T7 Express. The cells harboring pET21-CtDH were cultivated in small-scale with the auto-inducing MagicMedia to optimize the expression conditions. Baffled shaken flasks (250 mL) were filled with 78 mL MagicMedia and 2 mL of overnight seed culture (1:40 dilution) and were incubated at 30 °C in a shaking incubator (130 rpm) until an OD_600_ > 6.0 was reached. Then the temperature was reduced to 25 °C for 18 h more. Cells were harvested by centrifugation, resuspended in Tris–HCl buffer (50 mM, pH 7.5; 0.5 M NaCl), and disrupted by ultrasonication. After centrifugation, the enzymatic activity of the supernatant was compared using the DCIP assay.

*Aspergillus niger* cotransformation was carried out as previously described by Punt and van den Hondel [41], using the pAN52-CtCDH and pAB4-1 in a 10:1 ratio. Transformants were selected for uridine prototrophy on selective solid minimum medium (without uridine) and incubated for 10 days. In order to screen the best transformant for enzyme production in liquid medium, 50 mL of culture medium was inoculated with 2 × 10^6^ spores mL^−1^ in a 250-mL baffled flask. The culture was monitored for 14 days at 30 °C and 130 rpm. The pH was adjusted to 5.5 with 1 M citric acid and activity was checked daily.

*Trichoderma reesei* transformation was performed as described [42] using uncut plasmid DNA. Transformants were streaked twice onto PDA plates that contained 50 µg mL^−1^ of hygromycin B, and purified by plating conidiospores onto PDA plates with 0.1% Triton X-100 as colony restrictor. Candidate transformants were screened in shaking flasks (160 rpm, 28 °C) in a modified MA-Medium (50 mL in a 250-mL shaken flask) for 10 days. The pH was adjusted daily to 5.0 with 2 M KOH. Culture supernatants were collected by filtration with Miracloth and used for enzymatic analysis.

### Production of recombinant *Ct*DH/*Ct*CDHs in different systems

An overnight seed culture of the *E. coli* transformant (selected from a LB plate with 100 μg mL^−1^ ampicillin) was inoculated at a 1:40 dilution into 6 L MagicMedia (which starts the expression of *Ct*DH by autoinduction) with 100 μg mL^−1^ ampicillin in a 7-L glass vessel fermenter (MBR Bioreactor, Wetzikon, Switzerland). The initial cultivation temperature was 30 °C, the variable airflow rate was around 6 L min^−1^, and the agitation was set to 500 rpm. After 6 h, the cultivation temperature was changed to 25 °C. Samples were taken initially and after 3, 6, 11, 24 and 28 h. Cells were collected by centrifugation and used to determine wet biomass. The pellet was resuspended in 50 mM Tris–HCl buffer and disrupted by ultrasonication. The intracellular CDH activity and protein concentration were assayed after centrifugation. The corresponding specific activity of the crude extract was calculated from the volumetric activity of the crude extract divided by the specific activity of the purified enzyme.

*Aspergillus niger* fermentation was carried out in 7-L MBR fermenter, inoculating 8 × 10^9^ spores in 4 L culture medium. The following set points were used: pH 5.5, T = 30 °C, Airflow 6 L min^−1^. The pH was maintained using 2 M citrate acid. Initial agitation speed was 350 rpm. At day 5, agitation was increased to 600 rpm and 0.5 L water was added to submerge the mycelium. Samples were taken daily and the CDH activity, protein and glucose concentration of the supernatant were measured.

Cultivation of *T. reesei* was performed in a Sixfors bioreactor (Infors HT, Bottmingen, Switzerland) with a working volume of 0.4 L. Inoculum culture was pre-grown in 250 mL Erlenmeyer flasks on a rotary shaker (160 rpm) at 28 °C, containing 50 mL modified MA-medium. Pre-culture grown for 24 h was filtered, the biomass washed with sterile water and transferred into the fermenter. Operating conditions were: pH 5.0, 28 °C, 400 rpm and 0.4 vvm (volumes of air per volume of liquid per minute). Samples were taken daily and activity was measured.

Enzyme production with each expression system was repeated twice (biological duplicates). The expression of volumetric activity and protein concentration differed by less than 10% (data not shown). We only report the results of the fermentation from which we purified the enzyme. All measurements of volumetric activities and protein concentrations were performed three times (technical triplicates). Protein concentrations were determined by the Bradford method using a prefabricated assay from Bio-Rad Laboratories and bovine serum albumin (BSA) as the calibration standard [43]. Glucose concentrations were measured by d-glucose Assay Kit (Megazyme, Wicklow, Ireland).

### Purification of recombinant *Ct*DH/*Ct*CDHs in different systems

The *E. coli* fermentation broth was centrifuged at 4000×*g* for 10 min at 4 °C, the pellets were suspended in 1.5 L Tris–HCl buffer (50 mM, pH 7.5; 0.5 M NaCl) and disrupted using a APV Rannie und Gaulin homogenizer. The crude extract obtained by centrifugation (6000×*g*, 30 min, 4 °C) was loaded onto a HiTrap HP column (65 mL, GE Healthcare). The His-tagged *Ct*DH was eluted by a linear gradient of imidazole (5–500 mM) in Tris–HCl buffer (50 mM, pH 7.5; 0.5 M NaCl). Active fractions (~60 mL) were pooled and desalted using a HiTrap Desalting Column. The enzyme solution was further purified and concentrated 10-fold using an ultrafiltration module (30-kDa cutoff, Sartorius Stedim, Germany).

The purification strategy for the supernatants of *A. niger* and *T. reesei* fermentations was similar as previously described [15]. The culture broth was clarified by filtration with Miracloth and centrifuged at 6000×*g* for 20 min. Saturated ammonium sulfate solution was gently added to give a 20% saturation and particles were removed by centrifugation (6000×*g*, 30 min, 4 °C). The clear supernatant was applied on a Phenyl FF hydrophobic column (70 mL, GE Healthcare) equilibrated with 50 mM sodium acetate buffer pH 5.5 containing 20% (saturation) ammonium sulfate. The proteins were then eluted by a linear gradient from 20 to 0% (NH_4_)_2_SO_4_ in the same buffer in 5 column volumes at a flow rate of 1 mL min^−1^. Absorbances at 280, 420 (haem *b*), and 450 nm (FAD) were measured online along with the conductivity values. Fractions were collected automatically and tested by the cyt *c* activity assay. Active fractions were pooled and then diafiltered with sodium acetate buffer using an ultrafiltration module with a 10-kDa cutoff until a conductivity of 2–3 mS cm^−1^ was obtained. The partially deionized enzyme solution was loaded on a Q15-source anion exchange column (19 mL, GE Healthcare), previously equilibrated with 50 mM sodium acetate buffer pH 5.5. Proteins were eluted by increasing the amount of elution buffer (50 mM sodium acetate buffer, pH 5.5, containing 500 mM NaCl) linearly from 0 to 100% in 10 column volumes at a flow rate of 0.5 mL min^−1^. Fractions containing CDH activity were pooled and concentrated by ultrafiltration through a polyether–sulfone membrane with a 10 kDa molecular mass cutoff (Vivaflow crossflow cassette, Sartorius, Les Ulis, France). The purity and molecular weight of the recombinant enzymes were determined using SDS-PAGE.

### Electrophoretic analysis

SDS-PAGE was carried out by using Mini-Protean TGX precast gels (Bio-Rad Laboratories, Austria) with a gradient of 4–15% polyacrylamide. Proteins were visualized by Coomassie brilliant blue staining. The molecular mass under denaturating conditions was determined with a Precision Plus Protein Dual Color Standard (Bio-Rad Laboratories, Austria). All procedures were done according to the manufacturer’s recommendations.

To estimate the degree of glycosylation, homogenous enzymes were treated with N-glycosidase F (PNGase F) (New England BioLabs, Frankfurt, Germany), which cleaves the bond between N-Acetyl-glucosamine and Asparagine in N-linked glycoproteins under denaturing conditions, according to the manufacturer’s instructions. Reaction mixtures were incubated for 1 h at 37 °C and analysed on SDS-PAGE gels.

### UV/Vis spectra and determination of FAD loading

Spectra of homogeneously purified proteins were recorded from 700 to 250 nm at room temperature in both the oxidized and reduced states using a Hitachi U-3000 spectrophotometer (Hitachi, Tokyo, Japan). The purified proteins, which were in the oxidized state after purification, were diluted in 50 mM sodium acetate buffer, pH 5.5, to an absorbance at 280 nm of ~1 before recording a spectrum. The spectrum of the reduced enzyme was measured immediately after addition of a 100-fold molar excess of cellobiose to the cuvette. The molar absorption coefficients at 280 nm for all proteins were calculated by using the mature amino acid sequence and the ProtParam program (http://web.expasy.org/protparam/). The calculated molar absorption coefficients of *Ct*DH at 280 nm (ε_280_ = 109 mM^−1^ cm^−1^) and *Ct*CDH (ε_280_ = 149 mM^−1^ cm^−1^) were used for the determination of the protein concentration. To assess the purity of the various CDH preparations, the R_Z_ values, defined by the ratio A_420_/A_280_, were calculated for each CDH in its oxidized state.

The FAD loading was determined according to a published protocol using trichloroacetic acid to release the non-covalently bound FAD cofactor from the protein by precipitation [26]. After precipitation, the pH of the solution was carefully titrated with grains of solid sodium carbonate to a pH of 6–7. Then a spectrum was taken immediately to quantify the amount of FAD in the solution. The amount FAD was calculated using the molar absorption coefficient for free FAD (ε_450_ = 11.3 mM^−1^ cm^−1^).

### Enzyme activity assays

Enzyme activity was assayed spectrophotometrically using cyt *c* (ε_550_ = 19.6 mM^−1^ cm^−1^) as electron acceptor for intact CDH or DCIP (ε_550_ = 6.8 mM^−1^ cm^−1^) as an electron acceptor for both the intact holoenzyme and the dehydrogenase domain. The activities were determined by monitoring the reduction of 300 μM 2,6-dichlorophenol indophenol (DCIP) in 50 mM sodium acetate buffer (pH 5.5) containing 30 mM lactose and 4 mM of sodium fluoride (sodium fluoride was used as a laccase inhibitor). The cyt *c*-based assay contained 50 mM Tris–HCl buffer, pH 7.5, 20 µM cyt *c* and 30 mM lactose. The reaction was monitored for 180 s at 30 °C in a Lambda 35 UV–Visible spectrophotometer featuring a temperature-controlled 8-cell changer (Perkin Elmer, Massachusetts, USA). Enzyme activity was defined as the amount of enzyme that oxidizes 1 µmol of the electron acceptor per minute under the assay conditions.

### Steady-state-kinetic measurements

The kinetic parameters were determined for cellobiose oxidation measured at 30 °C in 100 mM McIlvaine buffer pH 5.5 using DCIP or pH 7.5 using cyt *c*. The concentration of cellobiose ranged from 10 μM to 5 mM with both electron acceptors (DCIP and cyt *c*). Triplicates were run to ensure reliable kinetic parameter determination. Catalytic constants were calculated using nonlinear least squares regression by fitting the observed data to the Michaelis–Menten equation (Sigma Plot 12.0, Systat Software). Protein concentration was determined by measuring absorbance at 280 nm (A280).

### Thermal stability measurements

*Thermo*FAD assays were performed according to Forneris et al. [27] and Reich et al. [44]. This method is based on the intrinsic fluorescence of the flavin cofactor of the flavoproteins and the fluorescence of the flavin cofactor is quenched by the protein environment. 25 µL of enzyme solution (15, 7.5 and 1.5 µM) was heated from 30 to 85 °C in increments of 1 °C per min using a MyiQ Real-Time PCR cycler (Bio-Rad Laboratories, California, USA) using an excitation wavelength range between 470 and 500 nm and an SYBR Green fluorescence emission filter (523–543), which falls within the same fluorescence range as the isoalloxazine ring of the FAD (470–570). The *T*_m_ values were obtained as the maximum of the first derivative of the sigmoid curves and the reported *T*_m_ values are the mean value of 12 independent experiments (triplicate for each enzyme concentration).

Alternatively, unfolding of the intact CDH was monitored by following the intrinsic tryptophan fluorescence upon heating [45]. Tryptophan is located on both CYT and DH (from 21 Trp residues 5 are located in CYT and 16 are located in DH). Experiments were performed in a temperature controlled Cary Eclipse fluorescence spectrophotometer (Agilent Technologies, California, USA) at a total volume of 2 mL and CDH or DH concentration of 1.5 µM. Cuvettes were stirred throughout the experiment. A heat ramp from 30 to 85 °C was applied at a rate of 1 °C per min. The excitation wavelength was 279 nm; the emission was recorded at 320 and 360 nm. The *T*_m_ was determined by fitting the tryptophan fluorescence emission ratio of 360–320 nm to a sigmoidal function. All experiments were performed in triplicates.

## Additional file

**Additional file 1: Table S1.** Purification schemes of recombinant *Ct*DH/*Ct*CDH. **Table S2.** Comparison of recombinant DH domain and intact CDHs from literatures. **Figure S1.** SDS-PAGE of recombinant *Ct*DH expressed in *E. coli*. **Figure S2.** Thermostability of *Ct*DH/*Ct*CDHs measured by the tryptophan fluorescence (A-D) and the *Thermo*FAD method (E-H).

## Abbreviations

CDH: cellobiose dehydrogenase
*Ct*CDH: CDH from *Corynascus thermophilus*
CtDH: dehydrogenase domain of *Ct*CDH
CYT: cytochrome domain of CDH
cyt *c*: cytochrome *c*
DCIP: 2,6-dichloroindophenol
DET: direct electron transfer
MET: mediated electron transfer
DH: dehydrogenase domain of CDH
FAD: flavin adenine dinucleotide
IET: intramolecular electron transfer
LPMO: lytic polysaccharide monooxygenase
