# Supplementary material for: Molecular and catalytic properties of fungal extracellular cellobiose dehydrogenase produced in prokaryotic and eukaryotic expression systems
Source: Microb Cell Fact. 2017 Feb 28;16:37. doi: 10.1186/s12934-017-0653-5 (PMC5331742; doi:10.1186/s12934-017-0653-5)
Supplement: Supplementary file 1 — Additional file 1: Table S1. Purification schemes of recombinant CtDH/CtCDH. Table S2. Comparison of recombinant DH domain and intact CDHs from literatures. Figure S1. SDS-PAGE of recombinant CtDH expressed in E. coli. Figure S2. Thermostability of CtDH/CtCDHs measured by the tryptophan fluorescence (A-D) and the ThermoFAD method (E-H). [file 12934_2017_653_MOESM1_ESM.docx]

**Table S1** Purification schemes of recombinant *Ct*DH/*Ct*CDH. Enzyme activity is measured with the DCIP assay for the DH domain and the cyt *c* assay for CDH. The protein concentration of *Ct*DH and *Ct*CDH in brackets was measured photometrically by using their molar absorption coefficients at 280 nm (*Ct*DH ε_280_ = 109 M^-1^ cm^-1^, *Ct*CDH ε_280_ = 149 M^-1^ cm^-1^). Specific activities in brackets were based on these values.

| ***Ct*DH expressed in *E. coli*** | | | | | |
| --- | --- | --- | --- | --- | --- |
| Purification step | Total activity (U) | Total protein (mg) | Specific activity (U/mg) | Yield (%) | Purification (fold) |
| Crude extract | 4434 | 10469 | 0.42 | 100 | 1 |
| IMAC | 3971 | 163.2 | 24.3 | 89.5 | 57.9 |
| Ultrafiltration | 2654 | 96.4 (135.7) | 27.5 (14.8) | 59.8 | 65.5 |
| ***Ct*CDH expressed in *P. pastoris*** [15] | | | | | |
| Purification step | Total activity (U) | Total protein (mg) | Specific activity (U/mg) | Yield (%) | Purification (fold) |
| Culture supernatant | 1179 | 3150 | 0.37 | 100 | 1 |
| Homogenous CDH (re-measured after 3 years of storage at -80°C) | 837 | 239 (442.6) | 3.48 (1.97) | 71 | 7.7 |
| ***Ct*CDH expressed in *A. niger*** | | | | | |
| Purification step | Total activity (U) | Total protein (mg) | Specific activity (U/mg) | Yield (%) | Purification (fold) |
| Culture supernatant | 114 | 579 | 0.19 | 100 | 1 |
| Phenyl Sepharose | 90.4 | 44.6 | 2.03 | 79 | 10,3 |
| Q source | 62.3 | 5.61 (11.2) | 11.1 (5.56) | 54 | 51.8 |
| ***Ct*CDH expressed in *T. reesei*** | | | | | |
| Purification step | Total activity (U) | Total protein (mg) | Specific activity (U/mg) | Yield (%) | Purification (fold) |
| Culture supernatant | 458 | 124 | 3.69 | 100 | 1 |
| Phenyl Sepharose | 334 | 51.6 | 6.48 | 73 | 1.7 |
| Q source | 265 | 21.2 (54.6) | 12.5 (5.59) | 58 | 3.4 |

**Table S2** Comparison of recombinant DH domain and intact CDHs from literatures.

| Fungal producer of CDH | Expression host | Expressed Volumetric DCIP activity, U L^-1^) | Expressed recombinant CDH conc., mg L^-1^) | Purification yield (%) | Purification fold | Specific activity (DCIP assay, U mg^-1^) | Specific activity (Cyt c assay,U mg^-1^) | Degree of glycosylation (%) | Reference |
| --- | --- | --- | --- | --- | --- | --- | --- | --- | --- |
| *Phanerochaete chrysosporium* (DH) | *E. coli* | 386 | 39 | 2 | 48 | 9.9 | - | 0 | [18] |
| *Phanerochaete chrysosporium* (CDH I) | *P. pastoris* | 1800^*^ | 79 | n.g. | n.g. | n.g. | n.g. | 6.5 | [19] |
| *Pycnoporus cinnabarinus* (CDH I) | *P. pastoris* | 7800 | 351 | n.g. | n.g. | 22.2 | 3.9 | 10 | [14] |
| *Trametes versicolor* (CDH I) | *P. pastoris* | 5218^*^ | 0.46^**^ | 20 | 16.7 | n.g. | 17300^**^ | 27(46) | [33] |
| *Corynascus thermophilus* (CDH II) | *P. pastoris* | 376 | 92 | 71 | 7.7 | 4.1 | 2.84 | 14-23 | [15] |
| *Myriococcum thermophilum* (CDH II) | *P. pastoris* | 2150 | 290 | n.g. | n.g. | 7.4 | n.g. | 23.5 | [34] |
| *Neurospora crassa* (CDH IIA) | *P. pastoris* | 1700 | 80 | 73 | 6.4 | 21.2 | 8.3 | 29 | [21] |
| *Neurospora crassa* (CDH IIB) | *P. pastoris* | 410 | 80 | 11 | 15 | 5.1 | 3 | 48 | [21] |
| *Thielavia terrestris* (CDH II) | *A. oryzae* | n.g. | n.g. | n.g. | n.g. | n.g. | n.g. | 3.9 | [24] |
| *Humicola insolens* CDH II | *A. oryzae* | n.g. | n.g. | n.g. | n.g. | n.g. | n.g. | n.g. | [35] |
| *Coprinopsis cinerea* CDH I | *A. niger* | 7600 | 108 | n.g. | n.g. | 70 | n.g. | 3.8 | [16] |
| *Podospora anserina* CDH II | *A. niger* | 126 | 20 | n.g. | n.g. | 6.2 | n.g. | 3.8 | [16] |

^*^activity was measured by cyt *c* assay; n.g. data not given; - DH domain has no cyt *c* activity. ^**^the reported values are unlikely and have to be considered cautiously.


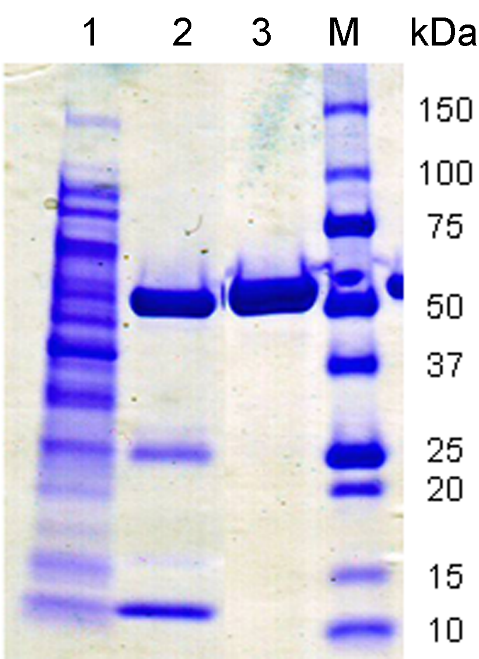


**Figure S1** SDS-PAGE of recombinant *Ct*DH expressed in *E. coli*. Lane M, molecular mass standard; lane 1, crude extract; lane 2, partially purified *Ct*DH after IMAC; lane 3, purified *Ct*DH after ultrafiltration.

**

**

**Figure S2** Thermostability of *Ct*DH/*Ct*CDHs measured by the tryptophan fluorescence (A-D) and the *Thermo*FAD method (E-H). The results of *Ct*DH/*Ct*CDHs expressed in *E. coli* (A, E), *P. pastoris* (B, F), *A. niger* (C, G) and *T. reesei* (D, H) are shown.
